# Multidisciplinary therapy strategy of precision medicine in clinical practice

**DOI:** 10.1002/ctm2.15

**Published:** 2020-03-31

**Authors:** Mengjia Qian, Qian Li, Miaomiao Zhang, Xiaojing Xu, Qi Shen, Hao Chen, Xiangdong Wang, Tianshu Liu, Yunfeng Cheng

**Affiliations:** ^1^ Zhongshan Hospital Institute of Clinical Science Fudan University Shanghai Medical College Shanghai China; ^2^ Department of Oncology Fudan University Shanghai Medical College Shanghai China; ^3^ Department of Thoracic Surgery Zhongshan Hospital Xuhui Branch Fudan University Shanghai Medical College Shanghai China; ^4^ Center for Tumor Diagnosis & Therapy Jinshan Hospital Fudan University Shanghai Medical College Shanghai China; ^5^ Department of Hematology Zhongshan Hospital Fudan University Shanghai Medical College Shanghai China

**Keywords:** cancer, gene sequencing, MDTS‐PM, precision medicine, target therapy

## Abstract

The application of precision medicine concept in clinical work needs a period of practice and experience accumulation. The present article introduced an example of functioning approach named “multidisciplinary therapy strategy of precision medicine” (MDTS‐PM), clinical practice and process, decision‐making, and therapies. The MDTS‐PM integrates multidisciplinary experts and develops real‐time therapeutic strategy based on clinical phenomes and gene sequencing of tissue DNA and circulating DNA. The strength of MDTS‐PM is the combination of dynamical clinical phenomes, genetic information, diagnosis, and treatment to make the therapy more targeted and specific. MDTS‐PM provides comprehensive, whole‐process, and personalized diagnosis and treatment services for patients with complex cancer or complex drug resistance progression; provides guidance for further adjustment of drug use; and establishes a multidisciplinary cooperative team, improves the quality of clinical diagnosis and treatment, and optimizes the process of medical services.

## 1 INTRODUCTION

Precision medicine is an emerging discipline of prevention and treatment strategies to translate the molecular approach into precise target therapy for inherited genetic disorders and cancers.^1^ Using clinical trans‐omics to integrate clinical phenomes with molecular multi‐omics, disease‐specific biomarkers and therapeutic targets can be identified and validated to find the causes of diseases and improve precise diagnosis, treatment, and prevention for specific patients.^2^ Based on comprehensive molecular phenomes and characterizations of lung cancer (eg hereditary and somatic gene changes, mutation, and heterogeneity), we developed target‐driven therapies and strategies and proposed the precise self‐validation system named “Zhongshan strategy of precision medicine” as one of precision medicine approaches.^3^ The proposed strategy suggested to treat patients according to cancer gene mutations and heterogeneity, after the validation of target therapy in the patient's own cancer cells or in patient‐derived xenografts using their own cancer cells. The current article presents an approach of clinical precision medicine named “multidisciplinary therapy strategy of precision medicine” (MDTS‐PM) to provide comprehensive, whole‐process, and personalized diagnosis and treatment services for patients with cancer, and improve the level of clinical diagnosis and treatment as well as the quality of medical services.

The clinical practice of MDTS‐PM is fully dependent upon the needs of patients in disease diagnosis and treatment. The MDTS‐PM team includes the experts from different disciplines, for example, oncology, general medicine, radiology, pathology, biochemistry, genetics, bioinformatics, surgery, and pharmacology, to combine multidimensional specialties and provide diagnosis and treatment together. More precision medical analysts help to interpret gene data, and assist doctors to formulate treatment plans. Therapeutic strategy of precision medicine was designated and discussed on the basis of clinical phenomic profiles, including patient complaints, signs, pathological imaging, biochemical measurements, radiomic profiles, and gene heterogeneity, copy number, and mutations. The present article introduced an example of MDTS‐PM functioning approach, clinical practice and process, decision‐making, and therapies.

## 2 COLLECTION OF CLINICAL AND MOLECULAR PHENOMES

According to the MDTS‐PM approach, the expert group received requests by doctors from different departments (such as departments of oncology, respiration, hepatobiliary surgery, etc) to recommend patients with gene sequencing reports. The important variant genes of patients were analyzed by the expert group to provide the suggestions about targeted drugs, chemotherapeutic drugs, and other appropriate therapies. The MDTS‐PM team established a real‐time function of data sharing and mining to collect clinical and molecular phenomes.

For example, a patient, 51‐year‐old male, was suffering from the onset of lower back pain for 2 weeks and had the first visit at the clinic in September of 2018. Multiple metastatic tumors in the thoracic 11‐sac1 intervertebral were demonstrated by magnetic resonance imaging (MRI). The patient smoked for more than 20 years, 20 cigarettes per day. His father had a kidney transplant for chronic nephritis and uremia, took oral immunosuppressants, and developed bladder cancer 10 years later. The Eastern Cooperative Oncology Group (ECOG) score was 1, body mass index 24.5 kg/m^2^, and numeric rating scale score 3. The image of positron emission tomography (PET)/computed tomography (CT) scan demonstrated multiple tumors in the dorsal segment of the lower lobe of the right lung, multiple lymph node metastases in the mediastinum and the right hilum, multiple glassy nodules in the upper lobe of the right lung, tumor in the brain, bilateral pleural effusion, thickened gastric wall in the pylorus of the stomach, and increased metabolism and multiple bone metastases in the whole body.

**FIGURE 1:**
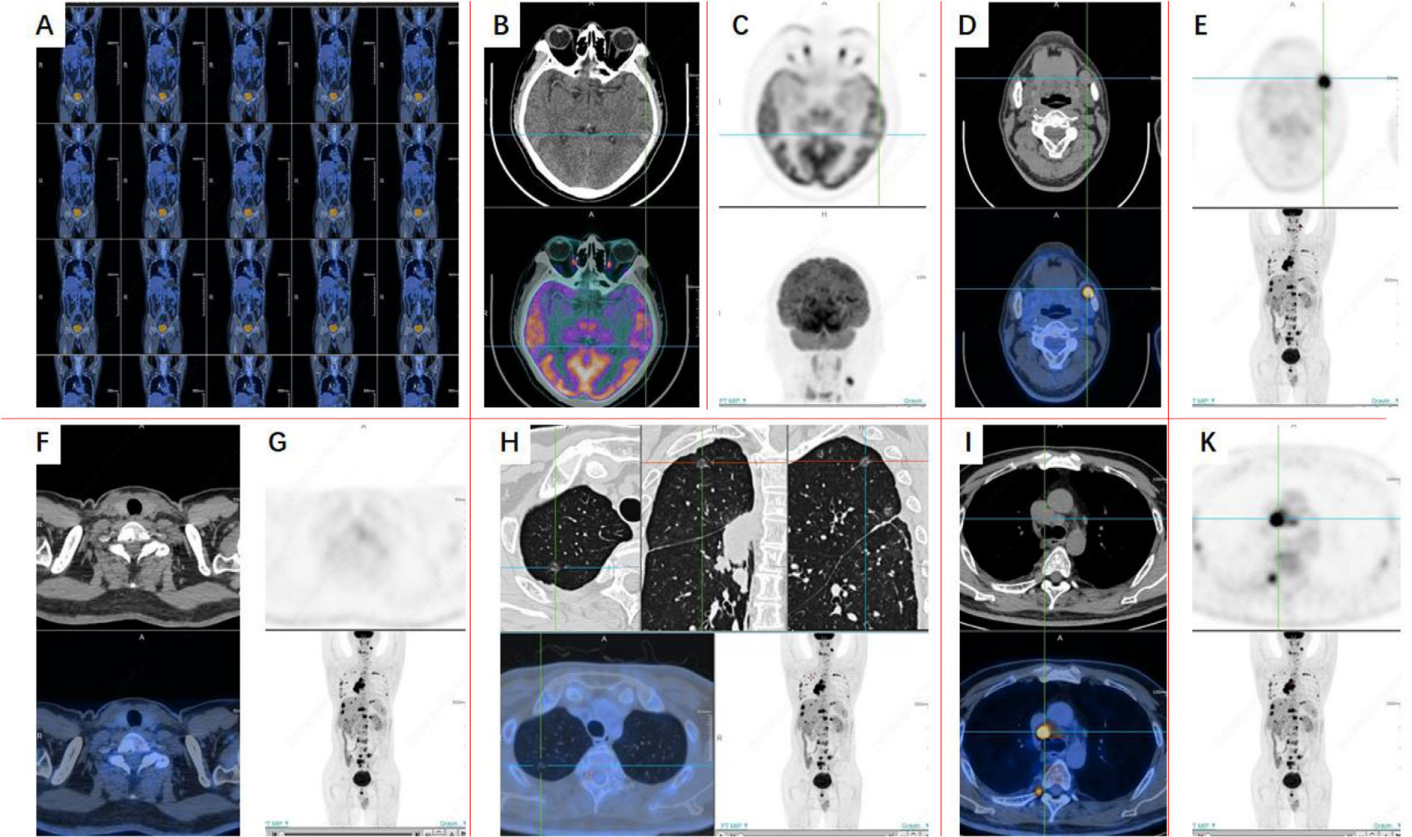
Imaging phenomes of positron emission tomography/computed tomography (PET/CT) 4 months after onset. After the treatment of lung MT with multiple systemic metastases, brain metastasis, left submaxillary lymph node metastasis, spleen and left adrenal gland metastasis, parathyroid lymph node metastasis is possible. A, Overview of PET/CT images. B‐E, PET/CT images of the brain. F and G, PET/CT images of the Pelvic. H‐K, PET/CT images of the chest

**FIGURE 2:**
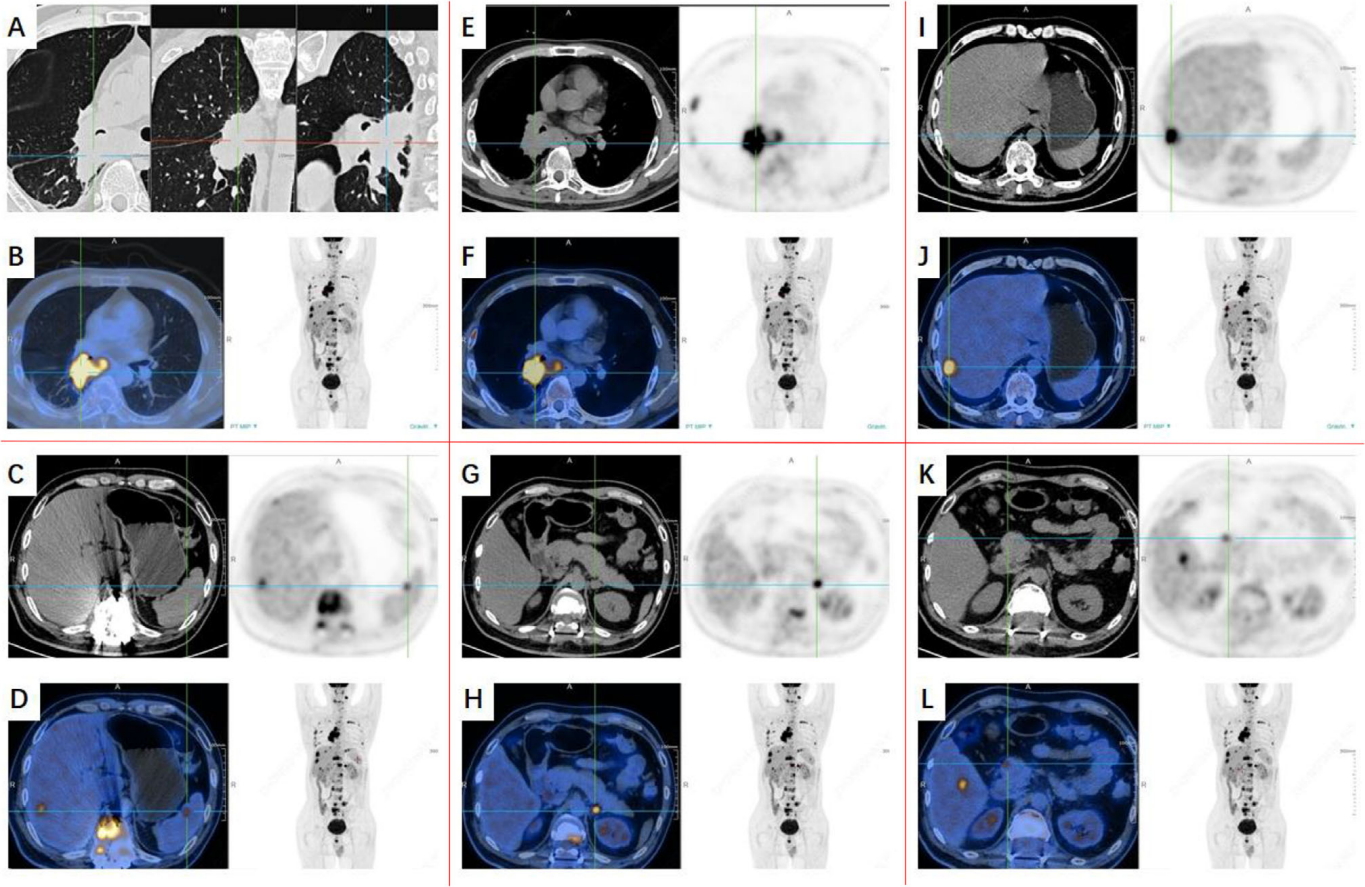
Imaging phenomes of positron emission tomography/computed tomography (PET/CT) 4 months after onset (continuation of Figure 1): Lung MT with multiple systemic metastases, more bone metastases and liver metastases, brain metastasis, left submaxillary lymph node metastasis, spleen and left adrenal gland metastasis. A,B,E,F, PET/CT images of the chest. C, D, and G‐L, PET/CT images of abdomen

Thoracolumbar resection and reconstruction of internal fixation were performed and pathology demonstrated metastatic large‐cell neuroendocrine carcinoma 1 month after onset, as detailed in Table 1. Epidermal growth factor receptor L858R mutation was defined by gene sequencing 2 months after onset, then gefitinib‐targeted therapy at a dose of 250 mg/day was started, and zoledronic acid bone repair therapy at 4 mg per time was given regularly. Patient felt a significant improvement of lower back pain and CT image showed the lesion was smaller in 3 months after onset. Nine days later, the patient's cough and pain in the ribs and hip bone became worse and cranial enhanced MRI showed multiple intracranial metastatic tumors. Four months after onset, the cough and bone pain of the patient aggravated. At this time, PET/CT images of the patient showed that the metastasis increased in multiple bones, liver, brain, left submaxillary lymph nodes, spleen, and left adrenal gland, during which the patient was diagnosed as a progressive disease. The overview of PET/CT images of the brain, pelvic, and chest are shown in Figure 1. PET/CT images of the chest and abdomen are shown in Figure 2.

**TABLE 1:** Immunohistochemical phenomes from staining of samples from lumbar vertebrae

**Biomarkers**	**P/N**	**Biomarkers**	**P/N**
Ki67	+ (65%)	P40	‐
NapA	‐	CK5/6	‐
CK7	+	CK20	‐
Villin	‐	CD56	+
CgA	‐	Syn	+
TTF‐1	+		

Abbreviations: CD56, cluster of differentiation 56; CgA, chromogranin A; CK5/6, cytokeratin 5/6; CK7, cytokeratin 7; CK20, cytokeratin 20; Ki67, antigen Ki67; NapA, NSF attachment protein alpha; P40, Np63; Syn, synapsin; TTF‐1, thyroid transcription factor 1; Villin, protein villin.

**FIGURE 3:**
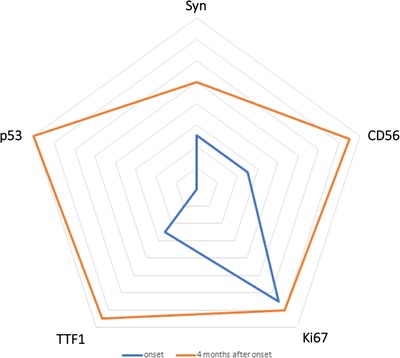
Changes of immunochemical staining results. The changes of immunochemical staining results including Syn, p53, CD56, TTF‐1, Ki67 are shown in the graph. The blue line shows onset results and the orange line shows results at 4 months after onset. The farther the lines away from the center, the more positive are the results

Poorly differentiated neuroendocrine carcinoma was observed and the density of immunochemical staining of positive markers increased from resected lung tissue to punctured tissue of right lung, for example, synapsin from + to ++, chromogranin A from negative to positive, cluster of differentiation 56 from + to +++, antigen Ki67 (Ki67) from 65 to 70%, thyroid transcription factor 1 from + to +++, and p53 from negative to 80%, whereas programmed cell death 1 (PD‐1), programmed cell death ligand 1 (PD‐L1) {28‐8}, and PD‐L1 {SP142} in tumor and interstitial tissues, ROS1, Braf V600E and EHRS, ALK, and SSTR5 were negatives (Figure 3). Table 2 demonstrates data of whole‐exome sequencing from resected lung tissue and peripheral blood after 1 month, and Table 3 presents 12 lung cancer–related genes from gene sequencing of circulating tumor DNA (ctDNA). Mutations of EGFR L858R, phosphatidylinositol 3‐kinase catalytic alpha polypeptide gene (PIK3CA) H1047R, Erbb2 receptor tyrosine kinase 2 (ERBB2) S310F, and retinoblastoma gene1 (RB1) were detected, rather than ALK, RET, ROS1, BRAF, HER2, c‐met, FGFR1, KRAS, TP53, PD‐1, PD‐L1, BIM, MEK1, VEGFR2, and VEGF. Table 3 demonstrates the chemosensitivity of cancer cells to platinum, etoposide, vincristine, 5‐fu, mitomycin, and methotrexate. The tumor mutational burden was less than 100 (Table 4). There is clinical problem for this patient that the tumor is pathologically mixed, adenocarcinoma and neuroendocrine carcinoma. What are the molecular characteristics of these two tumors? Which treatment should be given to the patient? MDT discussion is required.

**TABLE 2:** Categories of functional clusters, number and characteristics of altered genes, potential correlations with therapies, and list of key findings in the patient

**Categories**	**Number of genes**	**Genes**	**Key findings**
Key genes of lung cancer	17	EGFR, ALK, RET, ROS1, BRAF, HER2, PIK3CA, c‐MET, FGFR1, KRAS, TP53, PD‐1, BIM, MEK1, ALK, VEGFR2, VEGF	Three key gene mutations in lung cancer were detected
Immunotherapy	53	5	No mutations were detected
Tumor signaling pathway	–	–	Four abnormal tumor signaling pathways were detected
Family heredity	58	–	No mutations were detected
Others	25750	–	39 somatic cell mutations were detected
PD1/PDL1 signaling pathway	IFN signaling pathway JAK signaling pathway PD1/PDL1		No mutations were detected. Immunotherapy may have good effects on the patient.
MSI	Lynch syndrome–related genes (MSH2, MSH6, MLH1, PMS2)		No mutations were detected. Immunotherapy may not have good effects on the patient.
TMB	<100		Low TMB. Immunotherapy may not have good effects on the patient.

Abbreviations: ALK, anaplastic lymphoma kinase; BIM, BCL2 like 11; BRAF, B‐Raf proto‐oncogene, serine/theronine kinase; c‐MET, MET proto‐oncogene, receptor tyrosine kinase; EGFR, epidermal growth factor receptor; FGFR1, fibroblast growth factor receptor 1; HER2, human epidermal growth factor receptor‐2; IFN, Interferon; JAK, Janus kinase; KRAS, KRAS proto‐oncogene GTPase; MEK1, MAP kinase/ERK kinase 1; MLH1, mutL homolog 1; MSH2, mutS homolog 2; MSH6, mutS homolog 6; MSI, microsatellite instability.; PD‐1, programmed cell death 1; PIK3CA, phosphatidylinositol 3‐kinase catalytic alpha polypeptide gene; PMS2, PMS1 homolog 2, mismatch repair system component; RET, Ret proto‐oncogene; ROS1, ROS proto‐oncogene1 receptor tyrosine kinase; TMB, tumor mutation burden; TP53, tumor protein p53; VEGF, vascular endothelial growth factor; VEGFR2, vascular endothelial growth factor receptor

**TABLE 3:** Summaries of DNA sequence variations of the patients

**Gene**	**Type of mutation**	**Transcripts**	**Nucleotide change**	**Amino acid change**	**Mutation frequency**
PIK3CA	SNV	NM_006218	c.3240A>G	p.H1047R	52.81%
EGFR	SNV	NM_005228	c.2573T>G	p.L858R	35.78%
Gene fusion					
Gene	Loci	Variation ID			
Not detected					
Copy number variation					
Gene	Loci	Variation ID			
Not detected					

Abbreviations: EGFR, epidermal growth factor receptor; PIK3CA, phosphatidylinositol 3‐kinase catalytic alpha polypeptide gene; SNV, single nucleotide variation.

**TABLE 4:** List of selected target gene variations, variant allele fractions (VAF), and recommendations from MDTS‐PM

Gene	Variation	VAF (%)	Recommended drug
EGFR	L858R	24.35	Gefitinib, erlotinib, icotinib, afatinib, dacomitinib, osimertinib
PIK3CA	H1047R	24.14	Everolimus, tansimus, copanlisib
ERBB2	S310F	14.47	TDM‐1
RB1	E837	15.22	–
TMB<100	Low TMB	–	–

Abbreviations: ERBB2, Erb‐b2 receptor tyrosine kinase 2; EGFR, epidermal growth factor receptor; PIK3CA, phosphatidylinositol 3‐kinase catalytic alpha polypeptide gene; RB1, retinoblastoma gene1; TMB, tumor mutation burden.

## 3 DISCUSSION OF MDTS‐PM TEAM

The meeting of MDTS‐PM was held 4 months after the onset to discuss whether the patient had resistant genes against tyrosine kinase inhibitors (TKI), L858R can be used as a therapeutic target in this particular tumor, the chemotherapy regimen for non‐small cell lung cancer (NSCLC) or chemotherapy regimen for small cell lung cancer (SCLC) should be combined with targeted therapy, as well as radial and lumbar radiotherapy is required. The special concern was whether there was the most precise treatment for the patient with large‐cell neuroendocrine carcinoma. In this case, the neuroendocrine carcinoma was mixed with adenocarcinoma, more dangerous than adenocarcinoma alone and more difficult to be treated. The patient had the EGFR L858R mutation of adenocarcinoma, and developed the rapid drug resistance against TKI probably due to the combination of neuroendocrine carcinoma and adenocarcinoma. RB1 mutation is common in SCLC, while neuroendocrine cancer also has certain biological characteristics of SCLC.^4^ With the progression of the disease, mutations of EGFR L858R, PIK3CA H1047R, and ERBB2 S310F were detected in punctured lumbar tissues, and EGFR L858R and PIK3CA H1047R in ctDNA, consistent with the pathological characteristics of adenocarcinoma. It was proposed that the neuroendocrine carcinoma in the patient might be the type II pulmonary large‐cell neuroendocrine carcinoma, which is enriched for biallelic inactivation of TP53 and RB1, different from type I with biallelic TP53 and STK11/KEAP1 alterations.^5^ Mutations of EGFR and PIK3CA H1047R were considered to be associated with the rapid development of drug resistance and disease progression, as TKI treatment is not effective for patients with such mutation.^6^, ^7^ The patient also had HER2 mutations, which may also contribute to the rapid tumor cell growth by activating HER2‐encoded growth factor receptor‐related protein.

Considering the molecular mechanism, The PIK3CA inhibitor may improve PIK3CA mutation‐induced TKI resistance, as the patient had PIK3CA exon 20 mutation. However, the PIK3CA inhibitor is still in the clinical trials and is mostly used in breast cancer, so the clinical evidence for other tumors needs to be furthermore confirmed.^8^ The missense mutation of c.3140A>G;p.H1047R at 1047 of exon 21 of PIK3CA gene was detected in the patient, which was recorded for 2852 times in the Catalogue of Somatic Mutations in Cancer database, including 1632 times for breast cancer, 434 times for colorectal cancer, 183 times for endometrial cancer, 110 times for ovarian cancer, and 72 times for soft tissue sarcoma. PIK3CA as an isoform member of the PI3K family is a common somatic mutant oncogene with a high mutation rate in various tumors, such as 32% in colon cancer, 4‐25% in stomach cancer, 8‐40% in breast cancer, 5‐27% in meningeal cancer, 4‐25% in ovarian cancer, 11% in head and neck tumor, and 4‐33.1% in lung cancer, which were obtained by deeply mining data from the Catalogue Of Somatic Mutations In Cancer database (https://cancer.sanger.ac.uk/cosmic). Codon 1047 of PIK3CA gene is allocated in the PI3K/PI4K domain, where mutations can influence phospholipid kinase activity and activate the downstream of signaling pathways, altering the cell growth and survival.^6^, ^7^ Common mutations of PIK3CA, for example, E542K, E545K, and H1047R, could increase lipid kinase activity, activate the downstream AKT signaling pathway, and enhance tumorigenicity.^6^ It is challenging to define roles of PIK3CA mutations in the patients due to the complexity and specificity of PI3K isoform genes in transcriptional regulations and interactions during cell growth and proliferation.^9^ It is hard to clarify passive and negative regulations among PI3K‐associated target signal factors and select PI3K function‐specific inhibitors. For example, EGFR‐vIII mutation was found to negatively regulate gene expression of H2AZK4/7AC, H3K27AC, and ubiquitin‐specific protease 11 through the PI3K/AKT‐histone deacetylase2 axis.^10^

RB1, a tumor‐suppressor gene and negative regulator of cell cycle, regulates phosphoprotein in the nucleus through PB1‐encoded protein that contains n‐terminal, A/B pocket, and c‐terminal domains. Of those, the n‐terminal domain plays a key role in the inhibition of tumor growth through the P16/CyclinD/CDK4/RB/E2F pathway, during which dephosphorylation or hypophosphorylation of RB binds to intracellular transcription factor (E2F) and inhibits gene transcription.^11^ Mutation in RB1 gene losses the activation, prevents cells from S phase, and leads to the occurrence of lung cancer. There is no effective drug treatment for patients with RB1 gene mutation clinically.

p53 Mutations exist in most of the cancers, responsible for cancer cell resistance to drugs, which depends upon properties of anticancer drugs, biological functions of therapeutic targets, and mechanisms of interactions between drugs and targets. It was suggested that p53 mutations could alter molecular networks, dysregulate p53‐associated signal pathways, and cause drug resistance–associated clinical manifestations.^12^ We noticed that the degree of p53 mutation in the patient increased with the development of target drug resistance, indicating the urgent need of new targeted antitumor drugs and strategies, to improve the efficacy of the therapy. The experimental evidence showed that p53 mutation per se could cause probably the secondary changes in chemical structures and properties of PI3K subunit proteins or in interactions and intercommunications between p53 and PI3K isoform genes.^13^ The p53‐dependent cell sensitivity varied among target specificities, drug chemical properties, mechanism‐specific signal pathways, and drug efficacies. In this patient, we found that drugs induced the occurrence of new p53 mutations and increased the number of p53 mutation, probably leading to the real‐time occurrence of cancer cell resistance to a period treatment with drugs. We believe that comutations of p53, PIK3CA, and PB1 may play an important role in the development of drug resistance in the patients and be a new breakthrough point for new therapeutic strategy.

## 4 NOVEL TREATMENTS RECOMMENDED BY THE MDTS‐PM TEAM

One of the critical missions from MDTS‐PM team provides the recommendation of the new therapeutic strategy in clinical practice. For example, RB1 gene mutation was detected in the patient with neuroendocrine carcinoma and adenocarcinoma by gene sequencing, indicating the biological characteristics of small‐cell lung cancer. Etoposide and cisplatin (EP) chemotherapy was recommended in this stage. On basis of the high abundances and co‐existence of EGFR L858R and HER2 mutation as one of the adenocarcinoma properties in the patient, the combination of dual inhibitors targeting EGFR and HER2 tyrosine kinases was recommended. The targeted therapy of second‐line afatinib (40 mg/day) was also applied, as accompanied with local radiotherapy and analgesic treatment. Dynamic monitoring of ctDNA sequencing, especially those mutated genes detected, for example. EGFR and HER2 genes, was highly recommended. Since the gene sequencing reports are always complicated, for example, the team received another patient with EGFR L858R detected in tissue while not detected in blood, then how to choose the next treatment option for him? Therefore, the MDTS‐PM team will have lots to do in clinical practice.

## 5 FOLLOW‐UP FROM MDTS‐PM

**FIGURE 4:**
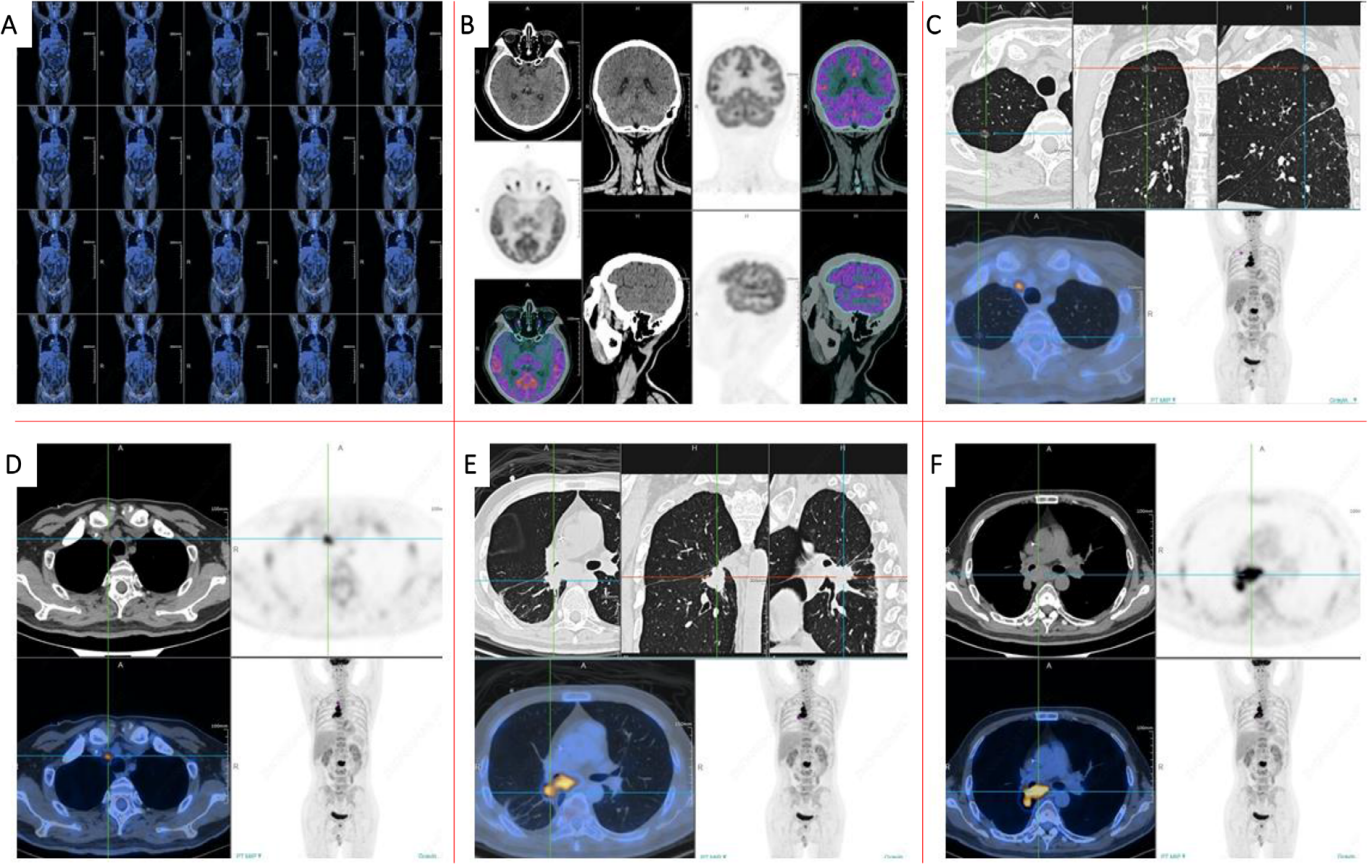
Imaging phenomes of positron emission tomography/computed tomography (PET/CT) 9 months after onset. Compared with the PET/CT images of 4 months after onset, the lesions in the lower lobe of the right lung were smaller than before. The glucose metabolism in the lymph nodes of the right upper mediastinal increased. The remaining diseased lymph nodes and left adrenal metastases were significantly reduced and glucose metabolism was significantly decreased. The tumor activity of brain, liver, and spleen metastases was inhibited. Multiple bone metastases all over the body were improved, the second lumbar tumor metabolism was active, and the glucose metabolism in residual bone disease foci was significantly decreased compared with the previous one. A, Overview of PET/CT images. B, PET/CT images of the brain. C‐F, PET/CT images of the chest

**FIGURE 5:**
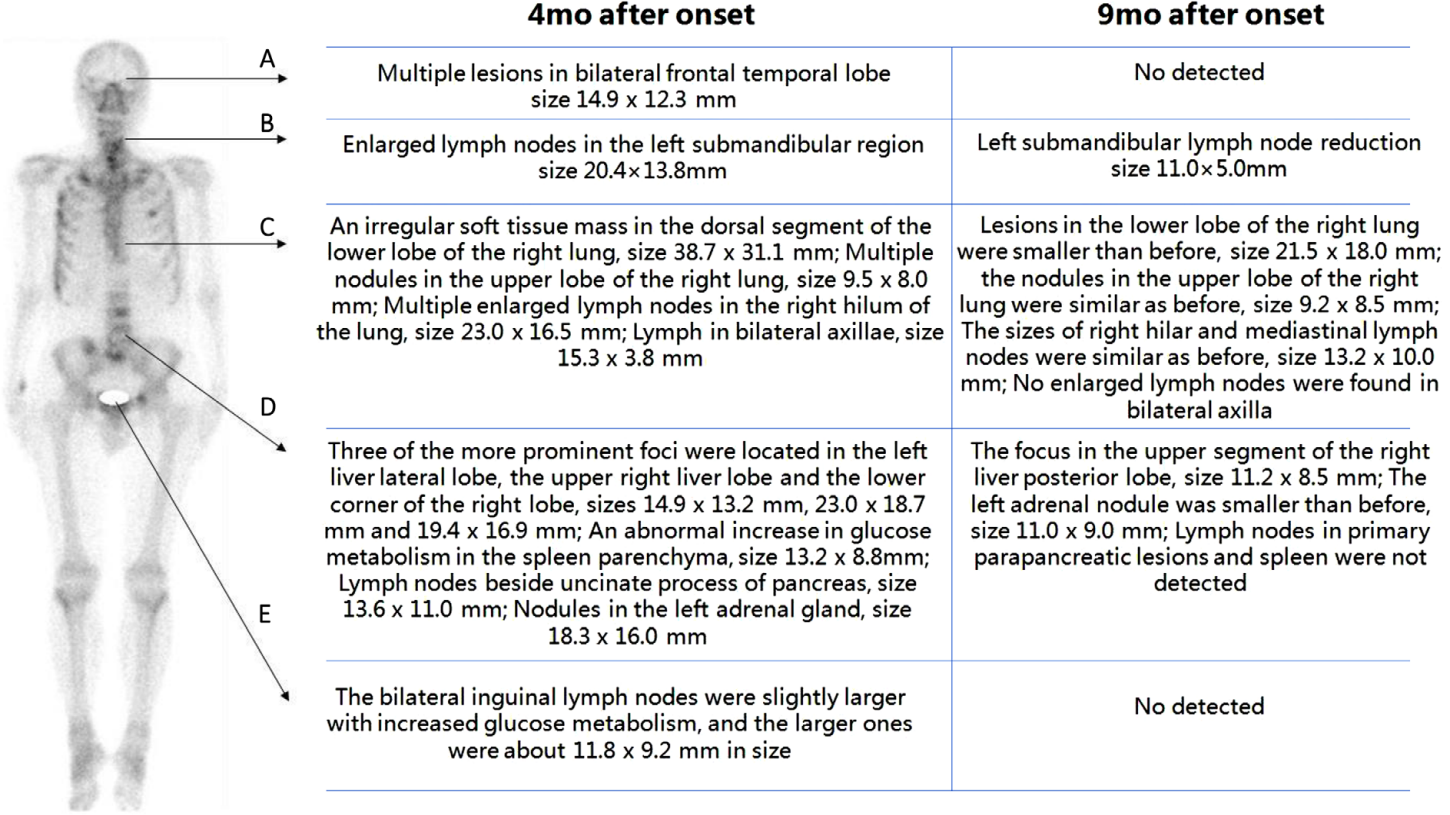
Changes of PET/CT results. The changes of PET/CT results including brain, neck, chest, abdomen, and pelvic are shown in the graph. After treatment, the images of 4 months after onset compared with the results of 9 months after onset, most lesions were smaller than before, or even not detected

**FIGURE 6:**
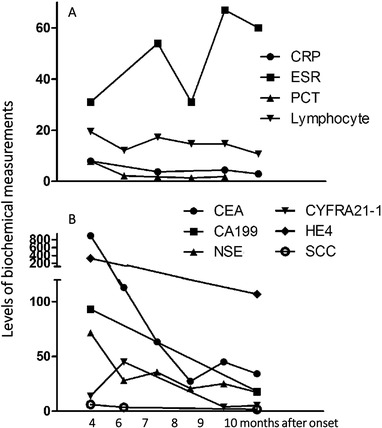
Changes of inflammatory and tumor markers. A, Changes of inflammatory markers including CRP, ESR, PCT, and lymphocyte% after 4/5/6/7/8/9/10 months after onset. B, Changes of tumor markers including CEA, CA199, NSE, CYFRA21‐1, HE4, and SCC after 4/5/6/7/8/9/10 months after onset

The precise follow‐up and real‐time update of patient responses to therapies are a part of MDTS‐PM activities for efficient dynamic monitoring and therapy. For example, the patient was evaluated by PET/CT images 9 months after onset (Figure 4) and compared with the images of 4 months after onset (Figure 5), and PD and the lung puncture were performed when the neuroendocrine cancer was diagnosed 4 months after onset. In order to further treat the cancer, the patient was given palliative second‐line chemotherapy with the first‐cycle EP regimen: etoposide at 190 mg (100 mg/m^2^) on day 1‐3 with cisplatin 60 mg on day 1 and 40 mg on day 2‐3 (total 75 mg/m^2^). Tomotherapy (TOMO) treatment was performed 10 times for cranial metastases and the targeted therapy with afatinib (40 mg/day) was given to the patient. The patient felt pain in the buttocks, discomfort in the forehead, and poor appetite after 10 days with afatinib (40 mg/day) therapy. After that, the afatinib given to the patient was reduced to 30 mg/day. In order to promote the process of bone repair, zoledronic acid at 4 mg was given during the second‐cycle EP regimen treatment. Palliative second‐line third‐ and fourth‐cycle EP chemotherapy was performed for additional 2 months. Another 2 weeks later, http://contrast-enhanced CT imaging assessed the disease as stable disease. The value of CEA was 34.3 ng/ml 10 months after onset (Figure 6).

Mediastinal lymph nodes slightly enlarged and chest metastatic lesion sizes reduced 9 months after onset, and then the patient underwent the fifth cycle of EP regimen. The TOMO radiotherapy was conducted under the guidance of the chief physician 11 months after onset, during which the target field was set for L2 and a dose 2800 cGy was delivered in 10 fractions, and the support treatment was also given during radiotherapy. MRI of the brain showed multiple metastatic foci in brain, of which the largest one was more than 1.6 cm 13 months after onset. The third measurement of ctDNA demonstrated that EGFR L858 (16.16&)/21 exon mutation was 0.4% and ERBB2 mutation was 20%. Considering the high mutation rate of ERBB2, pyrrotinib (320 mg/day) was recommended to be added in the EP treatment. The clinical phenomes of the patient were stable until the current report.

In conclusion, the present report documented MDTS‐PM as an approach of clinical precision medicine in practice to integrate multidisciplinary experts and develop real‐time therapeutic strategy on the basis of clinical phenomes and gene sequencing of tissue DNA and circulating DNA.^13^ Based on the team experience, the state of MDTS‐PM was summarized in Table 5. Different from the treatment on basis of pathology, MDTS‐PT conducts genetic testing and analysis before choosing therapeutic drugs.^14^, ^15^ The strength of MDTS‐PM is the combination of dynamical clinical phenomes, genetic information, diagnosis, and treatment to make the therapy more targeted and specific. MDTS‐PM provides comprehensive, whole‐process, and personalized diagnosis and treatment services for patients with complex cancer or complex drug resistance progression; provides guidance for further adjustment of drug use, establishes a multidisciplinary cooperative team, improves the quality of clinical diagnosis and treatment, and optimizes the process of medical services.^16^

**TABLE 5:** Summary of the state of MDTS‐PM

**The state of MDTS‐PM**	
Patient	Complicated or drug resistant With gene sequencing results
Departments	Oncology, general medicine, radiology, pathology, biochemistry, genetics, bioinformatics, surgery, pharmacology, etc
Flow	Receive requests from different departments Collect the clinical phenome, gene sequencing results, and other relevant examination results Hold discussion with relevant experts of the team Give suggestions to the patient
Advantages	Professional in interpreting gene sequencing reports Guidance for further adjustment of medication One‐stop diagnosis and treatment
Challenges	How to adjust the medication in view of so many complex drug resistance mechanisms? How to better integrate clinical phenomes with molecular multi‐omics, disease‐specific biomarkers, and therapeutic targets?

## CONFLICT OF INTERESTS

The authors declare that there is no conflict of interest.

## GLOSSARY

**Table:** 

Term	Definition
Clinical phenomes	A series of abnormal changes in the body of a patient that include general signs, pathological manifestations, imaging manifestations, inflammatory indicators, and tumor markers.
Molecular phenomes	A series of changes in genes that include hereditary and somatic gene changes, mutation, and heterogeneity.
Multi‐omics	It includes proteomics, transcriptomics, miRNA omics, metabolomics, and lipidomics.
Precision medicine	Precision medicine is based on the internal biological information and clinical symptoms and signs of the patient to implement personal health care and clinical decision‐making for patients.
Target therapy	Treatment at the cellular and molecular level for known carcinogenic sites.
Trans‐omics	It includes multi‐omics, clinical phenomes, disease‐specific biomarkers, and therapeutic targets.
